# COVID-19 Pandemic and Changes in Children’s Behavioral Problems: The Mediating Role of Maternal Depressive Symptoms

**DOI:** 10.3390/children10060977

**Published:** 2023-05-31

**Authors:** Stacey N. Doan, Anna Beth Burniston, Patricia Smiley, Cindy H. Liu

**Affiliations:** 1 Department of Psychological Science, Claremont McKenna College, Claremont, CA 91711, USA; 2 Department of Population Sciences, City of Hope National Medical Center, Claremont, CA 91711, USA; 3 Department of Psychological Science, Pomona College, Claremont, CA 91711, USA; 4 Department of Pediatric Newborn Medicine & Psychiatry, Brigham and Women’s Hospital, Boston, MA 02215, USA

**Keywords:** COVID-19 mental health, depressive symptoms, internalizing behaviors, externalizing behaviors, family systems, stress proliferation

## Abstract

The COVID-19 pandemic has been linked to a range of behavioral problems in children. To date, however, longitudinal studies with data prior to the pandemic are rare, and moreover, few studies have examined the family context. This is notable as evidence suggests that mothers were highly vulnerable to the effects of the pandemic, and stress proliferation models would argue that children’s wellbeing are undoubtedly affected by maternal wellbeing. In the current investigation, we examine changes in maternal depressive symptoms and children’s behavioral problems from prior to the pandemic to the first few months of COVID-19 in the U.S. The results suggest a significant increase in children’s internalizing problems and maternal depressive symptoms. Consistent with stress proliferation models, the relationship between COVID-19-related stressors and children’s behavioral problems were mediated by maternal mental health.

## 1. Introduction

There is increased concern that the COVID-19 pandemic may be leading to psychological problems (e.g., depression, anxiety), even among children [1]. However, to date few studies have tested this hypothesis longitudinally with pre-pandemic baseline data. Indeed, the majority of research has only focused on concurrent data collected during the pandemic. The lack of longitudinal data severely limits our ability to draw conclusions about the impact of the pandemic on children’s outcomes. In the context of research on stress, the pandemic embodies several characteristics that make it unique. First, given its nature, there are elements of uncontrollability, as well as unknowability. Moreover, unlike stressors such as natural disasters, it is chronic and ongoing. All of these factors contribute to the fact that COVID-19 is a significant chronic stressor, with important implications for mental health. At the same time, there is significant variability in its impacts. Evidence suggests that the effect of the pandemic is variable with some individuals experiencing worst outcomes than others. This difference is likely attributable to individual personality characteristics, as well as the number of COVID-19 related stressors that an individual may experience [2].

While children have been mostly protected from the worst health impacts of the pandemic, a host of pandemic-related risks has been particularly salient to children’s psychological health. For example, school closures and the lack of extracurricular and social activities, as well as quarantining and social distancing requirements, deprived children of social interaction with their peers. Indeed, research has demonstrated higher levels of depressive symptomology and externalizing behavioral problems in preschool-aged children during the pandemic as compared to pre-pandemic norms [3]. Notably, however, the study also identified the home environment as a factor that mitigated the detrimental influences of the pandemic. In Ireland, where during the first lockdown individuals were prohibited from leaving their home except for essentials, qualitative data suggest that children were demonstrating symptoms of anxiety, depression, social isolation and behavioral problems [4]. A recent review paper concluded that the pandemic led to increased fear, depression and anxiety symptoms in children and adolescents, with older adolescents, girls and youth living with neurodiversity or physical health conditions most vulnerable [5]. While this body of work is compelling, it has several notable limitations. The majority of this work with children and adolescents is concurrent in nature, and few longitudinal data exist that track participants from the pre-pandemic period. Moreover, children’s development does not occur in isolation, and bioecological systems theory [6] suggests that children’s outcomes must be considered in the context of both micro- and macrosystems. Particularly relevant to the current research, we argue that to understand children’s well-being during the pandemic, we must consider the well-being of parents.

The impact of the pandemic on parents of young children is notable. A significant majority of parents reported experiencing sleep issues and symptoms of anxiety and depression during the current crisis [7]. Mothers’ mental health may be particularly impacted by the pandemic. Women are taking on a disproportionate amount of the childcare and homeschooling responsibilities, further widening the existing gender gap in domestic burdens. The impact of the pandemic on mothers is evident from research on academic professionals, with reports demonstrating significant reductions in the productivity of mothers of young children [8]. Women also constitute a large proportion of frontline healthcare workers, so mothers who are considered essential workers may experience anxiety about their heightened risk of transmitting COVID-19 to family members. Compounding on these stressors, women are at a greater risk of experiencing domestic abuse during stay-at-home orders [9].

Stress proliferation models suggest that psychological effects on children due to the pandemic may be primarily mediated through maternal mental health [10]. Mothers are experiencing new pandemic-related stressors, such as those working remotly without childcare, which could undermine their ability to respond supportively to their children’s distress. The effects of stress contagion may differ as a function of individual differences in coping abilities and underlying conditions, such as mental health issues (i.e., depression and anxiety) and being immunocompromised. Maternal depression may compromise mothers’ ability to engage in sensitive and supportive parenting behaviors [11]. 

Here, we examine changes in children’s behavioral problems and maternal depression, from data obtained approximately 1.5 years prior to the pandemic at time 1 (T1) and during the first wave of the pandemic (T2, May–October 2020), and consider their direct and indirect relation to COVID-19-related stressful events.

## 2. Methods

Families participated in a longitudinal study on parenting and child health. Baseline data were collected in the laboratory between 2017 and 2019 (T1); follow-up data were collected between May and October 2020 (T2). T2 assessments were conducted during the first wave of the pandemic (May–August 2020) through an online survey. The United States declared a national emergency on 13 March 2020. During May 2020, California was under a “stay-at-home” order. Schools and businesses were closed to in-person activities. During June 2020, most schools were still closed to in-person learning, but restaurants were allowed to open at reduced capacity. Toward the end of summer, in July and August, California had reimposed restrictions, schools remained closed and indoor activities including restaurants and entertainment venues were not allowed. Thus, at T2, participants were in one of the most restrictive phases of the pandemic. Mothers were paid $100 for their participation in the baseline study and $75 for the follow-up. Children received a small gift. The study was approved by Pomona College’s IRB on 29 June 2021. The case file is #04292016JB-MP.

### 2.1. Participants

We had 185 mother–child dyads who participated in the baseline. Of these, 152 dyads (M_age_ = 71.05) participated in the follow-up (83% retention). In the baseline, mothers with typically developing children, aged between 3–6 and English speaking participated. Of these, 152 dyads (M _child age_ = 71.05 months) participated at T2 (83% retention) during the early months of the pandemic between May and October 2020 (T2).

### 2.2. Adverse COVID-19-Related Events

At T2, mothers completed a 30-item Adverse COVID-19 Events questionnaire in which they indicated whether a stressor (e.g., lost a job, became sick from COVID-19, etc.) had occurred since the pandemic began. The frequency of events was tallied, and the sum was used for analyses. This questionnaire was based on prior assessments that assess exposure to stressful life events [12] and social readjustment [13]. In addition, items were modified and added specific to the pandemic (e.g., became sick from COVID-19, had difficulty with remote work). Sample items included “you tested positive for coronavirus/COVID-19” and “you lost your job due to the pandemic.” Cronbach’s α for the scale was acceptable at 0.70.

### 2.3. Maternal Depression

At T1 and T2, the Beck Depression Inventory [14] was used to assess depressive symptoms in mothers. For this study, the suicide item was removed given issues with risk management during the pandemic. The survey asked participants to self-report their depressive symptoms (e.g., feelings of sadness, guilt and disappointment) on a zero-to-three scale. Sum scores were used for analyses. Cronbach’s alpha was 0.93 and 0.91 at T1 and T2, respectively.

### 2.4. Child Behavioral Problems

At T1 and T2, mothers reported on children’s behavioral problems using the Child Behavior Checklist [15]. We used the two major broadband scales: internalizing problems and externalizing problems. Parents were asked to respond on a scale from 0 (not true) to 2 (very true or often true) to questions such as “my child is too fearful or anxious” and “my child destroys things belonging to his/her family or other children”. Cronbach’s α was 0.89 for internalizing and 0.92 for externalizing problems.

### 2.5. Statistical Analyses

Analyses were conducted using the LAVAAN package in R [16], which estimates missing data using full information maximum likelihood [17]. The inspection of variables was first conducted to examine their distribution. We first examined changes in children’s internalizing and externalizing problems from T1 to T2 using a dependent samples *t*-test. Next, we looked at changes in maternal depressive symptoms. Regression analyses were then used to examine the relationship between maternal depression and adverse events on children’s outcomes. Finally, we conducted a mediation analysis to examine the extent to which changes in maternal depression mediated the relationship between adverse events and child outcomes.

## 3. Results

**Figure 1:**
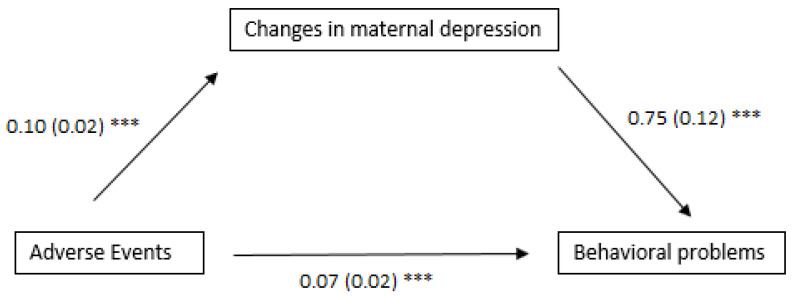
All models control for child sex, age, maternal education and baseline behavioral problems. Behavioral problems modeled also controlled for baseline levels. *** *p* < 0.001. Estimates and standard errors are provided with standard errors in parentheses. Estimates for the path from adverse events to children’s behavioral problems indicate the indirect effect.

There was a significant increase in children’s internalizing behaviors over time, *t*(184) = 5.13, *p* < 0.001, but no change in externalizing behaviors. Maternal depressive symptoms also increased from the baseline (*M* = 8.5, *SE* = 0.61) to T2 (*M* = 10.12, *SE* = 0.62), *t*(184) = 2.42, *p* = 0.02. Next, adverse events and standardized residual depression scores were regressed on children’s behavioral problems, controlling for maternal education, child age and sex, and T1 behavioral problems. Changes in maternal depression but not adverse events predicted internalizing, B = 3.04, *SE* = 0.5, *p* < 0.001, and externalizing behaviors, B = 2.92, *SE* = 0.57, *p* < 0.001 (See Table 1). Bootstrapping analyses [18] were used to test the indirect effect of adverse events as mediated by maternal depression on children’s outcomes. Given the interrelatedness of both internalizing and externalizing behavioral problems, they were standardized and summed to create a composite score. Mediation analyses demonstrated that there was a significant main effect of adverse events on changes in maternal depressive symptoms and a significant effect of changes in maternal depression and children’s overall behavioral problems. The indirect effect was also significant, *B* = 0.07, *SE* = 0.02, *p* < 0.001 (Figure 1).

We also wanted to disentangle the same models for the specific subset of behavioral problems (e.g., internalizing and externalizing behaviors) for the sake of completeness. The indirect effect was significant for both internalizing, B = 0.27, *SE* = 0.09, *p* = 0.002, 95% CI [0.27, 0.12], and externalizing, B = 0.28, *SE* = 0.08, *p* = 0.001, 95% CI [0.13, 0.45], behaviors, suggesting that the effects of adverse events were mediated by changes in maternal depression. As a sensitivity analysis, we also reran our analyses with the subset of participants who had complete data, and our results were replicated.

## 4. Discussion

In the current paper, we examine changes in both maternal and child outcomes from pre- to post-pandemic. Importantly, we took a family system framework and argue that children’s responses to the pandemic must be considered in the context of the family unit. The results of our study suggest an increase in maternal depression and children’s internalizing behaviors during the COVID-19 pandemic but not children’s externalizing behaviors. Importantly, changes in mothers’ depression levels predicted change in both types of problem behaviors. Moreover, adverse life events associated with the COVID-19 pandemic were indirectly associated with children’s behavioral problems through maternal depression, rather than exerting direct effects on the children themselves. In other words, maternal mental health mediated the relationship between adverse events and child outcomes.

Our research demonstrates that the pandemic has implications for the well-being of young children. At the same time, however, it is important to note that children who are most vulnerable are those whose parents are experiencing increased stress. Indeed, our data showed that the pandemic led to an increase in depressive symptoms in mothers, and it was this change in mental health that drove changes in children’s behavioral problems. When parents are stressed or anxious, they are more likely to engage in harsh or hostile parenting behavior [19]. This harsh and hostile parenting has consequences on child outcomes. It is important to note that, in addition, parents do not need to be hostile but rather those who are experiencing higher rates of depressive symptoms due to adverse events may simply be more likely to be unresponsive or emotionally unavailable to their children. This lack of sensitive responding is also detrimental to children’s development. Our research demonstrating the indirect effect of maternal depressive symptoms on child behavioral problems is consistent with past research showing parents’ perceived impact of COVID-19-related stressors decreased parent–child closeness and increased harsh parenting behaviors [20]; it is likely that parents’ decreased ability to provide sensitive care is the central driver of the impact of COVID-19 stressors on children’s outcomes.

Indeed, spillover and crossover effects based on stress proliferation models [10] highlight the importance of taking into account a family system model for prevention and intervention efforts. Maternal mental health has been inordinately impacted during the pandemic, with women taking on a disproportionate amount of childcare and homeschooling responsibilities [21]. Women also constitute a large proportion of frontline health care workers and also are at a greater risk of domestic abuse during stay-at-home orders [22]. These factors have made women, mothers particularly, vulnerable to pandemic effects. Given that the frequency and intensity of pandemics are likely to increase in the future, our data suggest that we must take a holistic context and consider the important role of mothers when examining children’s well-being. In sum, in considering how the effects of the pandemic might affect children’s outcomes and potential efforts to ameliorate detrimental consequences, it is imperative that researchers consider the family unit.

Despite the strengths of our study, including the longitudinal approach, there are several limitations. One important limitation of the study is we assessed depression and behavioral problems relatively early in the pandemic (approximately 5 months from March when the U.S. declared a national emergency). Despite the longitudinal nature of the study, we only captured one time point during the pandemic. Specifically, early on, when stress was most high, and challenges were novel and threatening. Over the course of the pandemic, it is likely that individuals began to adjust to the new normal. We underscore the importance of simultaneously tracking mother and child psychological well-being over the continued course of the pandemic, as well as identifying the moderators of these associations to inform intervention and prevention efforts. Indeed, there is a great need to understand how maternal depressive symptoms as well as children’s psychological adjustment may change over the course of a chronic stressor such as the pandemic.

Other limitations included our relatively small sample size, which made our data difficult to generalize. In our study, we controlled for maternal education as a proxy for socio-economic status, but socio-economic status may be an important moderator of our findings. Specifically, the results may be exacerbated in families from a lower socio-economic class. Relatedly, we did not consider race and ethnicity. Certain communities of color may be inordinately impacted. In addition, the pandemic led to an increase in discrimination against Asian people, specifically Chinese Americans, and this is another stressor not captured in our study. Moreover, it is important to note the significant variability in how countries and cities dealt with the pandemic. California was one of the most restrictive states in America, and thus, generalizing to other states and countries must be done with caution. Finally, our study also used self-report measures, and studies using observer ratings of children’s behavior would improve validity.

Regardless of these limitations, the longitudinal nature of the study and the assessment of specific stressors, rather than the assumption of stress during COVID-19, are strengths of the current study. Furthermore, our mediation model allows us to understand specific mechanisms that can be addressed to improve the well-being of children during pandemics. In considering factors that may promote well-being in children, our data suggest the utmost importance of policies that support the mental health and well-being of mothers. Specifically, social support, childcare and other resources (e.g., educational or financial) may ease the burden on mothers and improve their capacity to deal with stressors and consequently the well-being of their children. Finally, in our study, we focused on mothers given that they are often the primary caregivers of young children. However, many children live in a household with multiple adults, including fathers, grandparents and other relatives in addition to their mothers. The presence of these other adults may ameliorate the direct impact of the pandemic on children but also buffer the effects of stress on maternal outcomes. Considering the nuances of these family dynamics would be central for designing policies and interventions that help children weather any future pandemics. In sum, children’s outcomes must be considered within the context of micro systems such as the family, as well as macro-level (country) variables.

## Figures and Tables

**Table 1:** Main Effects Model Predicting Child Internalizing and Externalizing Behavioral Problems.

Child Internalizing Behavioral Problems						Child Externalizing Behavioral Problems				
Variable	Estimate	*SE*	z-Value	95% CI		Estimate	*SE*	z-Value	95% CI	
				Lower	Upper				Lower	Upper
BP ^a^	0.61 ***	0.09	6.70	0.43	0.78	0.44 ***	0.07	6.43	0.31	0.57
ALEQt ^b^	−0.26	0.16	−1.62	−0.57	0.06	0.05	0.17	0.28	−0.28	0.38
Age	0.03	0.04	0.82	−0.04	0.10	0.06	0.04	1.37	−0.02	0.13
Sex	−2.95 **	0.98	−3.01	−4.86	−1.03	−2.96 **	1.03	−2.87	−4.97	−0.94
Education	−0.05	0.39	−0.13	−0.81	0.71	−0.93 *	0.41	−2.27	−1.74	−0.13
BDIr ^c^	3.04 ***	0.54	5.60	1.97	4.10	2.92 ***	0.57	5.10	1.80	4.04

Note: ^a^ baseline behavioral problems, ^b^ total adverse events related to the pandemic, ^c^ standardized residuals of maternal depression scores from T1 to T2, * *p* < 0.05 (two-tailed), ** *p* < 0.01 (two-tailed) and *** *p* < 0.001.

## Data Availability

Data for the current study can be obtained by contacting the first author at 850 N Columbia Ave. Claremont Mckenna College, Department of Psychological Science, Claremont, California, 91711, United States of America.
